# Pulsed Optical Vortex Array Generation in a Self-Q-Switched Tm:YALO_3_ Laser

**DOI:** 10.3390/ma17051144

**Published:** 2024-03-01

**Authors:** Luyang Tong, Changdong Chen, Yangjian Cai, Lina Zhao

**Affiliations:** 1 Shandong Provincial Key Laboratory of Optics and Photonic Device, College of Physics and Electronics, Collaborative Innovation Center of Light Manipulation and Applications, Shandong Normal University, Jinan 250358, China; 2020010065@stu.sdnu.edu.cn; 2 College of Physics, Nanjing University of Aeronautics and Astronautics, Nanjing 211100, China; cdchen@nuaa.edu.cn; 3 Joint Research Center of Light Manipulation Science and Photonic Integrated Chip of East China Normal University and Shandong Normal University, East China Normal University, Shanghai 200241, China

**Keywords:** optical vortex arrays, pulsed laser, Tm:YAP, self-Q-switched laser

## Abstract

Optical vortex arrays are characterized by specific orbital angular momentums, and they have important applications in optical trapping and manipulation, optical communications, secure communications, and high-security information processing. Despite widespread research on optical vortex arrays, the 2 μm wavelength range remains underexplored. Pulsed lasers at 2 μm are vital in laser medicine, sensing, communications, and nonlinear optic applications. The need for 2 μm-pulsed structured optical vortices, combining the advantages of this wavelength range and optical vortex arrays, is evident. Therefore, using just three elements in the cavity, we demonstrate a compact self-Q-switched Tm:YALO_3_ vortex laser by utilizing the self-modulation effect of a laser crystal and a defect spot mirror. By tuning the position of the defect spot and the output coupler, the resonator delivers optical vortex arrays with phase singularities ranging from 1 to 4. The narrowest pulse widths of the TEM_00_ LG_0,−1_, two-, three-, and four-vortex arrays are 543, 1266, 1281, 2379, and 1615 ns, respectively. All the vortex arrays in our study have relatively high-power outputs, slope efficiencies, and single-pulse energies. This work paves the way for a 2 μm-pulsed structured light source that has potential applications in optical trapping and manipulation, free-space optical communications, and laser medicine.

## 1. Introduction

Optical vortices, characterized by a spiral phase front represented by exp(*−ilφ*) and carrying the orbital angular momentum (OAM) of the *lħ* per photon (where *l* denotes the topological charge number and *φ* denotes the azimuthal angle), have received widespread attention in the fields of optical trapping [1,2,3], optical communication [4,5], quantum entanglement [6,7], optical machining [8,9], and optical imaging [10,11]. Compared with vortex beams with conventional doughnut structures, optical vortex arrays (OVAs) are characterized by independent phase singularities and individual topological charges. Around the phase singularities, the intensity of the light is zero. Benefitting from these properties, OVAs have attracted widespread attention in the fields of optical trapping and manipulation [12,13], optical communications with a large capacity and high-security information processing [14], Bose–Einstein condensate states [15], optical modulation [16], etc. Therefore, a number of technologies have been gradually reported to generate OVAs. The common approach to generating optical vortices or OVA beams is through the use of phase modulation elements outside the laser cavity, such as spatial light modulators [17], spiral phase plates [18], and mode converters by means of cylindrical lenses [19]. However, the low damage threshold and diffraction loss of these optical-phase components impose limitations on high-power operations and high beam qualities. The Hermite–Gaussian (HG), Laguerre–Gaussian (LG), and Ince–Gaussian (IG) modes represent eigenmodes in laser resonators. OVAs have typically been described as the result of the coherence superposition of these eigenmodes [20]. By harnessing transverse mode locking, specialized laser resonators enable the creation of OVAs. The technique of directly generating optical vortex beams or optical vortex array beams within the cavity represents a significant innovation in the field of optics. Due to the absence of additional phase modulation elements, this method is capable of yielding optical vortex beams or optical vortex array beams with high efficiency and superior beam quality. Diverse methods were employed for the in-cavity generation of these beams, including the defect spots mirror, off-axis pumping, doughnut-shaped beam pumping, and a spherical aberration within the cavity. Each of these approaches provides a varied pathway for the direct generation of optical vortex beams. It not only has substantial theoretical and practical significance in the field of optics but also opens new possibilities for applications in related fields. Using doughnut-shaped beam pumping and the thermal lensing effect of a Nd:LYSO laser crystal, optical vortices with a changeable OAM were directly generated in the laser cavity in [21]. As the pump power increased, the transverse pattern of the laser changed from TEM_00_ LG_0,1_ to LG_0,2_. Through the combination of off-axis pumping and SESAM mode locking, femtosecond LG_01_ optical vortex beams with controlled chirality were achieved [22]. The theoretical research on obtaining an LG₀₁ vortex beam was grounded in the off-axis pumping scheme, leading to the generation of the diagonal HG₁₀ mode and the precise modulation of the Gouy phase difference between the HG₁₀ and HG₀₁ modes on both the sagittal and tangential planes. Picosecond optical vortex beams could be obtained by converting higher-order HG modes, which were generated from a Nd:GdVO_4_ self-mode-locked laser [23]. Using this technique, femtosecond optical vortex beams in the 2 μm region were achieved [19]. A femtosecond vortex beam with controlled chirality was directly generated in a Yb:KYW laser using an intracavity spot-defect mirror [24]. This method has also been used to generate ultrahigh-order LG modes. LG vortex beams of an adjustable order from 1 to 288 have been previously obtained by adjusting the ratio of the radius of the spot-defect pattern to the radius of the TEM_00_ mode on the output mirror [25]. The LG-mode vortex beam has been achieved by introducing spherical aberration within the laser cavity, obtaining LG modes up to the order of 317. Depending on the size of the different LG order modes within a cavity, one or a pair of plano-convex lenses have been inserted into the laser cavity to achieve the effect of mode selection [26].

Aside from the optical vortexes with one singularity, OVAs have been theoretically analyzed and experimentally achieved in a Na_2_ laser and in vertical-cavity surface-emitting lasers by means of the transverse mode-locking method [27,28]. In recent years, OVAs have also been achieved in microchips and thin-slice solid-state lasers [29,30,31,32,33,34]. A microchip laser with a large Fresnel number was used to generate optical vortex lattices by adjusting the output coupler’s reflectivity [29]. Nanosecond OVAs have been accomplished in a self-Q-switched (SQS) microchip laser [30]. From a laser diode end-pumped thin-slice solid-state laser, the controlled formation of radial and rectangular vortex arrays has been observed. Subsequently, the high-order HG modes and vortex arrays, by controlling the transverse distribution of pump light, have been further demonstrated [32]. Optical vortices with broadband and comb-like spectra have been generated in a continuous-wave Yb:YAG/YVO_4_ microchip laser by utilizing stimulated Raman scattering [33]. Optical vortex lattices in a thin-slice Yb:CALGO laser have been achieved by precisely controlling the pump aperture and off-axis displacement [34]. A range of OVAs with adjustable singularities, ranging from 1 to 10, has been generated within a microchip laser system by employing a tilted annular beam as the pump source [20]. We have directly generated OVAs in all-solid-state lasers operating at wavelengths of 1 μm and 3 μm by using the off-axis pumping method and introducing defect spots within the cavity [35,36]. However, to date, research on OVAs in the 2 μm wavelength range has received less attention. The 2 μm-pulsed lasers can be used in laser medicine [37], laser measurement and sensing [38], optical communications [39], and nonlinear optics [40] because they operate in the safe spectral region for the human eye, present strong absorption of water molecules, and are located in the atmospheric transmission window. There is an urgent need for 2 μm-pulsed structured optical vortices, as they combine the advantages of 2 μm-pulsed lasers and OVAs. Self-Q-switched lasers have simple and compact structures and play an important role in the generation of pulsed structured optical vortices.

Here, we report a compact laser that can directly generate 2 μm-pulsed vortex arrays. OVAs with phase singularities tunable from 1 to 4 were generated with a spot-defect mirror, which was fabricated using femtosecond laser-inscribing technology. The pulse was obtained via the self-modulation effect of the Tm:YALO_3_ (Tm:YAP) crystal. This work paves the way for the direct generation of 2 μm-pulsed structured optical vortices from a resonator, which may potentially be applied in optical trapping and manipulation, free-space optical communications, and laser medicine.

## 2. Materials and Methods

The Tm:YAP crystal, as a key player in the generation of the 2 μm laser, has received wide attention from researchers. As a natural birefringent crystal, the YAP crystal exhibits not only remarkable thermal conductivity but also intriguing optical properties. In our experimental setup, a 3% doped Tm:YAP crystal was used as the gain medium, and its absorption coefficient was 4 cm⁻^1^ for the 793 nm pump light. This crystal’s absorption bandwidth stretches impressively up to 30 nm, making it an ideal candidate in semiconductor-diode-laser-pumped all-solid-state lasers. Significantly, the Tm:YAP crystal boasts a strong emission with a cross-section of 0.73 × 10⁻^20^ cm^2^ and an emission spectrum of 200 nm. This distinctive feature amplifies its suitability for applications in the range of a wide spectrum. Furthermore, the Tm:YAP crystal’s upper-level lifetime is up to 4 ms, therefore, the long upper-level lifetime makes sure that the Tm:YAP crystal is an excellent candidate for generating high-energy pulsed lasers. These characteristics indicate that the Tm:YAP crystal is highly competitive in the field of high-power, high-energy lasers in the 2 μm wavelength region. 

Pulsed Tm:YAP lasers, based on self-Q-switching mechanisms, have received significant attention. Several explanations have been developed to clarify the inherent mechanism. Razdobreev et al. reported the self-Q-switching characteristic in a Tm:YAP microchip laser, attributing the self-pulse generation to the phonon-assisted excited-state absorption (ESA) mechanism [41]. However, Wu et al. demonstrated that this absorption cross-section is too weak to be the sole cause of self-pulsation, and they insisted the main reason was nonlinear dynamical chaos [42]. Cai et al. attributed this phenomenon to the presence of a time-dependent lensing effect within the gain medium and the thermal lensing effect arising from refractive index changes [43]. The more popular explanation is the ground-state re-absorption (GSRA) effect [44]. Tm:YAP is a quasi-three-level system. The crystal undergoes a transition of particles from the ground-state ^3^H_6_ to the excited states ^3^H_4_ and then ^3^F_4_ when exposed to pump light. Subsequently, excited-state particles transit back from ^3^F_4_ to the ground-state ^3^H_6_ through spontaneous emission and emit a photon at 1.91 μm. The excited-state particles could also absorb photons from ^3^F_4_ to a higher level_._ Due to the absorption cross-section of ground-state ^3^H_6_ being larger than that of the excited-state ^3^F_4_, the predominance of the above process is absorption. Tm^3^⁺ ions absorb the majority of photons at 1.91 μm, maintaining a relatively high absorption coefficient for photons and resulting in an increase in intracavity losses. With an increase in the pump light, the fluorescence is amplified. The majority of ground-state particles shift to the excited state, and the number of particles in a ground state that are capable of absorbing photons decreases. Consequently, the crystal becomes more transparent to photons, significantly increasing the quality factor of the resonant cavity. At this juncture, the dominant transition process is emission. Excited-state particles undergo avalanche transitions to the ground state under photon perturbation, generating a pulse. The crystal’s absorption coefficient for photons rapidly declines and intracavity losses escalate, eventually leading to the inability to sustain laser output. This cyclic process repeats, giving rise to self-Q-switched pulses.

**Figure 1:**
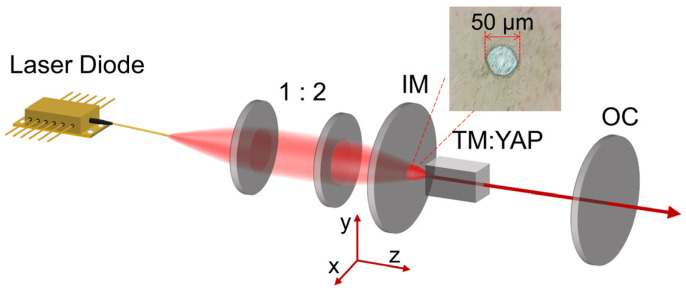
Schematic diagram of the experimental device for 2 μm-pulsed structured optical vortices lasers. The illustration is a microscopic image of a defect spot with a diameter of 50 μm.

The schematic of the experimental device for the 2 μm-pulsed OVAs is shown in Figure 1. The resonator is composed of a plane input mirror (IM) and a plano-concave output coupler (OC). The IM has a high transmissivity coated at 790–800 nm (T > 99%) and a high reflectivity coated at 1.9–2.1 μm (R > 99%). Additionally, on the right-hand side of the coated IM, we have fabricated several defect spots with diameters ranging from 30 to 400 μm using femtosecond laser-inscribing technology. The radius of the curvature of the OC is 100 mm, and it is coated with a 5% transmissivity at 1.9–2.1 μm. Both the IM and OC were mounted in a bracket that could be adjusted in three dimensions. The compact resonator had a total length of 43 mm and provided an applicable mode with a radius of approximately 170 μm at the IM. We selected a Tm:YAP crystal (3 × 3 × 10 mm^3^, c-cut, 3% doped) as the gain medium, and it was antireflection-coated (R < 1%) on the end surfaces at wavelengths of 793 nm and 1.9–2.1 μm. The pump source was a 793 nm laser diode array with a maximum output power of 50 W and a bandwidth of 3 nm, and the coupled fiber had a core diameter of 105 μm. Compared with a single-mode fiber, multimode fiber is advantageous due to its higher power and larger mode. For better mode overlap between the pump beam and cavity mode, the pump light was coupled to the laser gain medium through a 1:2 optical coupling lens. The intensity distribution and interference patterns of the pulsed OVAs were measured by a charge-coupled device (CCD) camera (Dataray, S-WCDLCM-IR-BB). An InGaAs detector (EOT, ET-5000) connected to a digital oscilloscope (LeCroy, HDO4104A) was used to record the pulse trains.

## 3. Results

**Figure 2:**
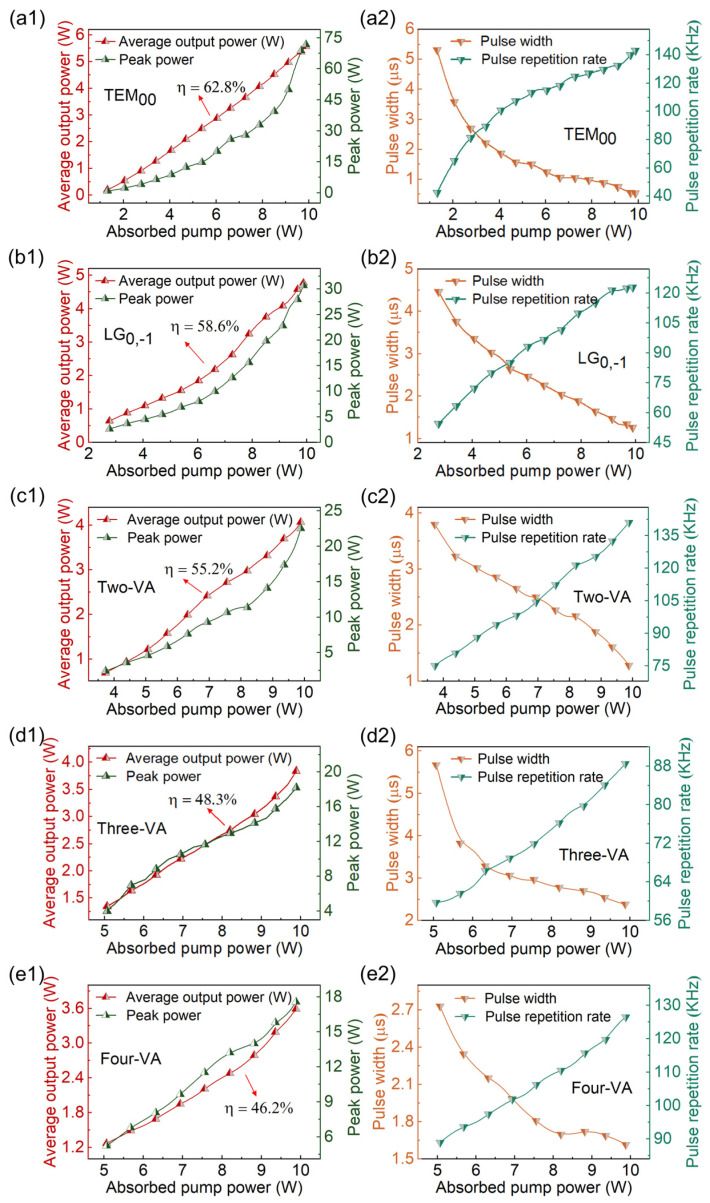
(**a1**–**e1**) Average output power and peak power and the (**a2**–**e2**) pulse width and repetition rate of different output modes as a function of absorbed pump power, respectively.

**Figure 3:**
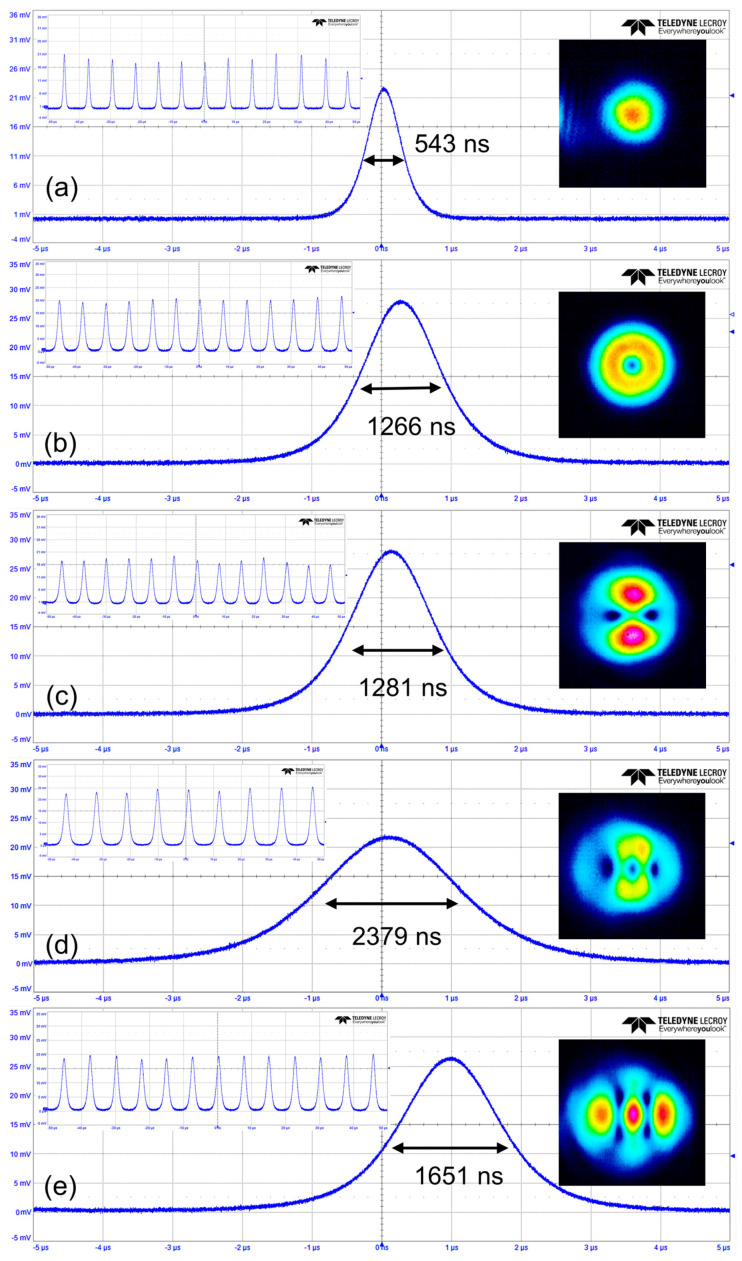
(**a**) TEM_00_, (**b**) LG_0,−1_, (**c**) two-VA, (**d**) three-VA, and (**e**) four-VA’s pulse trains with 1 μs/div; the insert is the pulse trains with 10 μs/div and intensity distributions of the different output modes at an absorbed pump power of 9.9 W.

In the experiment, the continuous-wave operation of the TEM_00_ mode was first achieved when the absorbed pump power exceeded 0.9 W. The absorbed pump power means the power that reaches the gain medium minus the residual pump power. Figure 2(a1,a2) provide the average output power, peak power, pulse width, and repetition rate of the TEM_00_ mode. In Figure 2(a1), when the absorbed pump power exceeds 1.3 W, the SQS TEM_00_ mode operation was obtained. The average output power and peak power increased linearly as the absorbed pump power improved. The slope efficiency of the average output power was 62.8%. The maximum average output power reached 5.6 W when the absorbed pump power improved to 9.9 W, and the corresponding single-pulse energy and peak power were 38.9 μJ and 71.7 W, respectively. As shown in Figure 2(a2), the pulse width shortened with raising the absorbed pump power. The pulse width reached the shortest 543 ns, while the corresponding maximum repetition frequency reached 143 kHz. Figure 3a shows the pulse trains at different timescales and intensity distributions of the output TEM_00_ mode. 

Different pulsed OVAs could be achieved by adjusting the position of the defect spot. All defect spots on the IM were tested. The resonator delivered lower output power and hardly generated a perfect four-vortex array if a larger defect spot was used. Therefore, the best one used in the experiment had a diameter of 50 μm, as shown in Figure 1. When the center of the defect spot coincided exactly with the optical axis, the LG mode with a doughnut shape was obtained, as shown in the inset of Figure 3b. The slope efficiency of the average output power of the LG mode was 58.6% (Figure 2(b1)). The maximum average output power, repetition frequency, and the narrowest pulse width were 4.8 W, 122.8 kHz, and 1266 ns, respectively. The highest single-pulse energy and peak power were calculated to be 38.8 μJ and 30.6 W (Figure 2(b2)), respectively. The position of the defect spot and OC in the transverse plane was defined as X_1_, Y_1_ and X_2_, Y_2_, respectively. Moving the OC’s position in the transverse direction results directly in lateral displacement from the axis, combined with the transverse displacement of the defect spot, thereby exciting diverse oscillation modes. When the defect spot was moved horizontally by 80 μm (X_1_ = 80 μm), a two-vortex array was obtained, as shown in the inset of Figure 3c. The slope efficiency of the two-VA was 55.2% (Figure 2(c1)). Under the absorbed pump power of 9.9 W, the maximum average output power was 4.07 W, the pulse width reduced to 1281 ns, and the corresponding maximum repetition frequency increased to 141 kHz. The corresponding peak power and single-pulse energy were 22.5 W and 28.8 μJ, respectively. As shown in the inset of Figure 3d, the pulsed three-VA was reported while the defect spot was shifted by 160 μm (X_1_ = 160 μm) and the OC by 50 μm (X_2_ = 50 μm) in the horizontal direction. The SQS three-VA’s threshold power was 5.1 W, as shown in Figure 2(d1). The slope efficiency was 48.3%, and the maximum output power was 3.84 W. The narrowest pulse duration and the maximum repetition frequency were 2379 ns and 88.5 kHz, respectively (Figure 2(d2)). The corresponding peak power and single-pulse energy were 18.2 W and 43.3 μJ, respectively. Through horizontally shifting the defect spot by 120 μm (X_1_ = 120 μm) and vertically by 90 μm (Y_1_ = 90 μm), and horizontally adjusting the OC by 70 μm (X_2_ = 70 μm), we observed a four-VA, as shown in the inset of Figure 3e. The pulsed four-VA started to oscillate when the absorbed pump power exceeded 5.1 W (Figure 2(e1)). The average output power of the four-VA reached a maximum of 3.59 W at the absorbed pump power of 9.9 W, with a slope efficiency of 46.2%. The pulse width was as short as 1615 ns, and the maximum repetition frequency was 126.5 kHz. Correspondingly, the peak power and single-pulse energy were 17.6 W and 28.3 μJ, respectively (Figure 2(e2)). Table 1 summarizes the parameters of achieving different pulsed vortex arrays.

**Figure 4:**
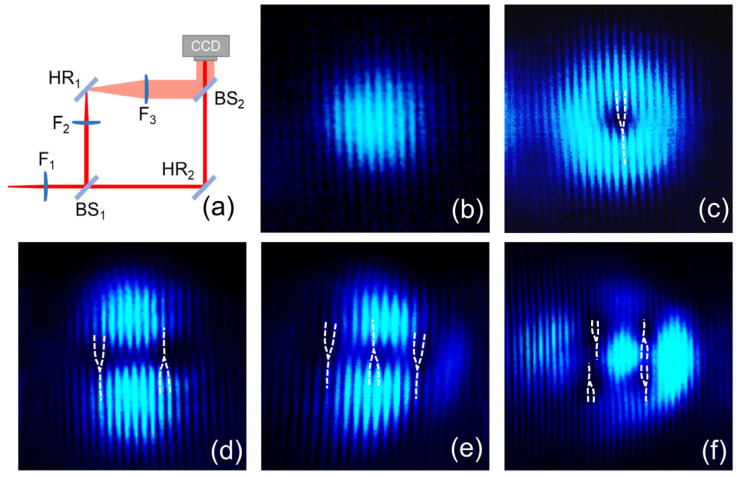
(**a**) The schematic of the homemade Mach–Zehnder interferometer. (**b**–**f**) The interference patterns of TEM_00_, LG_0,−1_, two-VA, three-VA, and four-VA, respectively.

The schematic diagram in Figure 4a depicts a homemade Mach–Zehnder interferometer. It consists of three plano-convex lenses (F_1_ = 300 mm, F_2_ = 25 mm, and F_3_ = 100 mm), two beam splitters (BS), and two high-reflectivity mirrors (HRs). The structured light beam was collimated by lens F_1_ and then split into two beams by BS_1_. One beam passed through a telescope system composed of lenses F_2_ and F_3_, where the beam was magnified. The two beams interfered with BS_2_. Using the CCD camera, the interference patterns of the pulsed OVAs were measured, as shown in Figure 4b–f. The fork-like interference pattern of the LG mode in Figure 4c indicates that the topological charge is *l* = −1. The topological charges of the two-VAs in Figure 4d are [−1, 1]. For the three-VA and four-VA, the topological charges are [−1, 1, −1,] and [−1, 1; 1, −1], as shown in Figure 4e and Figure 4f, respectively. Table 2 demonstrates that the maximum output powers and slope efficiencies of the pulsed OVAs decrease with the increase in the number of phase singularities, and the pulse widths become wider. This is primarily due to the greater loss caused by higher-order modes. The pulse width of the three-VA is larger than that of the four-VA. 

## 4. Discussion

According to the theory of transverse mode locking, the OVA is constituted by the superposition of different eigenmodes in the resonator [35]. The two-VA is generated by the superposition of LG_1,0_ and LG_0,±1_; the three-VA is the linear combination of four modes LG_1,±1_ and LG_0,±1_; and the four-VA is the superposition of three modes LG_1,0_ and LG_0, ±2_. Because the three-VA involves more transverse modes than four-VA, the pulse width of the three-VA is thus wider.

**Figure 5:**
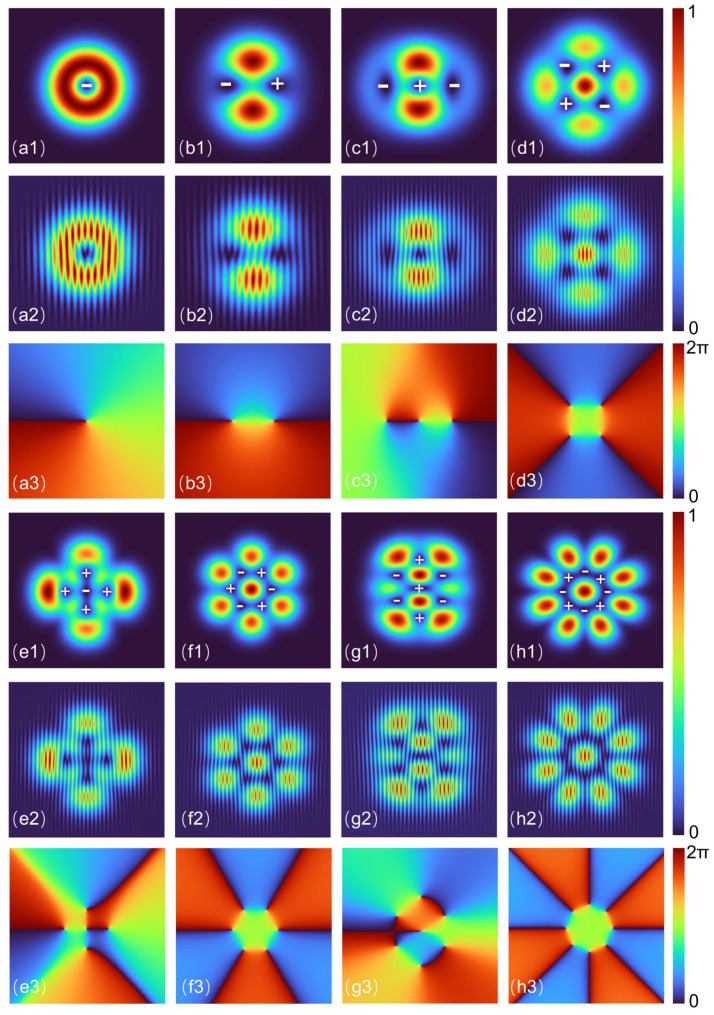
The theoretically simulated results of OVAs. (**a1**–**h1**) are transverse patterns; (**a2**–**h2**) are interference patterns; and (**a3**–**h3**) are phase distributions of optical vortex arrays with tunable phase singularities.

Here, the LG modes are chosen as the basic eigenmodes. The LG*_p_*_,*l*_ mode can be described as follows [35]:

(1) $$\begin{array}{l} M_{LG_{pl}}(r,\;\varphi ,\;\;z)=\sqrt{\frac{2p!}{\pi (p+\left|l\right|)!}}\;\;\times \frac{1}{W}{\left(\frac{\sqrt{2}r}{W}\right)}^{\left|l\right|}\exp\left(-\frac{r^{2}}{W^{2}}\right)L_{p}^{{\;}^{\left|l\right|}}\left(\frac{2r^{2}}{W^{2}}\right)\exp(il\varphi )\\ \;\;\;\;\;\times \exp\left[ikz+ik\frac{r^{2}}{2(z^{2}+z_{R}^{2})}-i(2p+\left|l\right|+1)\theta_{G}(z)\right] \end{array}$$

where *p* is the radial index and *l* is the angular index; $W=W_{0}\sqrt{1+{\left(z/z_{R}\right)}^{2}}$, $W_{0}$ is the radius of the fundamental mode’s beam waist; $z_{R}=\pi W_{0}^{2}/\lambda$ represents the Rayleigh length; $L_{p}^{{\;}^{\left|l\right|}}$ are the Laguerre polynomials; and $\theta_{G}(z)=\tan^{-1}(z/z_{R})$ is the Gouy phase. A stationary and complex vortex array is described as the superposition of a number of LG modes with different weights. For example, the two-vortex array is described as a linear combination of the three eigenmodes LG_1,0_, LG_0,1_, and LG_0,−1_. The respective weights attributed to these modes are uniformly set at 1. Additionally, their individual phases are 0, exp(iπ), and 0, correspondingly. In an analogous fashion, the three-vortex array is construed as a linear superposition involving four eigenmodes: LG_1,1_, LG_1,−1_, LG_0,1_, and LG_0,−1_. The designated weights for these modes are apportioned as 0.6, 0.6, 1, and 1, respectively. Additionally, their individual phases are specified as 0, 0, 0, and exp(iπ/2), correspondingly. Furthermore, the four-vortex array is constituted by LG_1,0_, LG_0,2_, and LG_0,−2_, with the respective weights allocated as 1.6, 0.8, and 0.8. The phases for these modes are calculated as 0, exp(iπ/2), and exp(iπ/2), respectively. Figure 5 demonstrates the simulated results, which represent the intensity, phase, and interference patterns of vortex arrays. Figure 5(a1–a3) depict the intensity pattern of the doughnut-shaped beam. Figure 5(a2,a3) illustrate the interference pattern and phase distribution of the LG mode doughnut-shaped beam, respectively. The presence of interference stripes with a singular fork in Figure 5(a2) and the clockwise phase change of 2π in Figure 5(a3) confirm the topological charge of the doughnut-shaped beam as −1. Figure 5(b2,b3) illustrate the interference pattern and phase distribution of the two-VA beam, respectively. In Figure 5(b3), the two-phase singularities exhibit a clockwise 2π phase change and a counterclockwise 2π phase change, respectively, proving that the topological charge of the two-VA beam is [−1, 1]. Figure 5(c2,c3) illustrate the interference pattern and phase distribution of the three-VA beam, respectively. In Figure 5(c3), the three-phase singularities undergo a clockwise, counterclockwise, and clockwise 2π phase change, respectively, establishing that the topological charge of the three-VA beam is [−1, 1, −1]. Similarly, the phase variations of the four-phase singularities in Figure 5(d3) indicate that the topological charges of the four-VA are [−1, 1; 1, −1]. Figure 5 illustrates that the simulations closely align with the experimental results, indicating a favorable agreement between the simulation and experimental results. Similarly, Figure 5(e1–h3) show the simulation results of the optical vortex arrays with 5−8 phase singularities, respectively. The five-vortex array is construed by three eigenmodes: LG_1,1_, LG_1,−1_, and LG_0,3_. The six-vortex array is composed of three eigenmodes: LG_1,0_, LG_0,3_, and LG_0,−3_. The seven-vortex array is the linear superposition of four eigenmodes: LG_1,1_, LG_1,−1_, LG_0,3_, and LG_0,−3_. The eight-vortex array is construed by three eigenmodes: LG_1,0_, LG_0, 4_, and LG_0,−4_. According to the simulation results, vortex arrays with higher order require multiple and higher-order eigenmodes. Experimentally, the generation of optical vortex arrays with phase singularities higher than four may require higher pump power, a more appropriate cavity mode size, and more precise control of the cavity.

Table 2 summarizes the parameters of various pulsed lasers in a 2 μm regime. Acousto-optic (A-O) Q-switching could achieve remarkably low repetition frequencies and high single-pulse energy, but it added complexity to the laser system. Furthermore, the work in reference [46] just reported the LG_0,1_ mode. Research on passive Q-switching, based on 2D material saturable absorbers (SA) and a SQS in a 2 μm regime, was mainly focused on the TEM_00_ mode. Unfortunately, more complicated vortex lasers, such as OVAs, were failed to demonstrate. Notably, in a simplified laser system, with the absence of modulation elements in the space and time domains, we obtained a relatively high output power of various pulsed OVAs. 

It is worth noting that, first, without additional modulation elements in the temporal domain, we obtained a SQS-pulsed laser according to the GSRA effect [44] inside the laser crystal. Second, the spot-defect mirror ensures that phase modulation and higher-order eigenmodes preferentially oscillate in the cavity. By tuning the position of the spot-defect mirror and output coupler, multiple eigenmodes are in phase and constitute OVAs by transverse mode locking. Therefore, with the absence of additional modulation elements in the temporal and spatial domains, the resonator delivers pulsed OVAs in a simplified system. Finally, the pulsed OVAs have relatively high power, high slope efficiencies, and high single-pulse energies in the 2 μm regime.

## 5. Conclusions

In conclusion, by just using three elements in the cavity, we demonstrated a compact SQS laser that can directly generate pulsed vortex arrays. The phase singularities of the optical vortex arrays are tunable from 1 to 4. All the optical vortex arrays have high-power outputs (greater than 3.59 μJ), slope efficiencies (greater than 46%), and single-pulse energies (greater than 28 μJ). The narrowest pulse widths of the TEM_00_, LG_0,–1_, two-VA, three-VA, and four-VA are 543, 1266, 1281, 2379, and 1615 ns, respectively. This work provides a method for a 2 μm-pulsed structured light source, which has potential applications in optical trapping and manipulation, free-space optical communications, and laser medicine.

## Figures and Tables

**Table 1:** Conditions for achieving different OVAs.

	X_1_ (μm)	Y_1_ (μm)	X_2_ (μm)	Y_2_ (μm)
LG_0,−1_	0	0	0	0
Two-VA	80	0	0	0
Three-VA	160	0	50	0
Four-VA	120	90	70	0

**Table 2:** Comparison of Q-switched laser with different output modes.

Laser Medium	Output Mode	Q-Switch Approach	Output Power (W)	Pulse Width (ns)	Repetition Rate (kHz)	Peak Power (W)	Single-Pulse Energy (μJ)	Ref.
Tm:YAP	TEM_00_	SQS	5.6	543	143	71.7	38.9	This work
Tm:YAP	LG_0,−1_	SQS	4.8	1266	122.8	30.6	38.8	
Tm:YAP	Two-VA	SQS	4.07	1281	141	22.5	28.8	
Tm:YAP	Three-VA	SQS	3.84	2379	88.5	18.2	43.3	
Tm:YAP	Four-VA	SQS	3.59	1615	126.5	17.6	28.3	
Tm:YAP	TEM_00_	SQS	1.68	1640	82.25	15.64	25.7	[43]
Tm:YLF	TEM_00_	SQS	0.61	1500	21	19.36	29	[44]
Tm:YAP	TEM_00_	SA	0.72	1090	82.25	7.11	8.86	[45]
Tm:LuYAG	LG_0,−1_	A-O	0.74	366	0.5	3800	1510	[46]

## Data Availability

Dataset available on request from the authors.
